# Enteral Nutrition in Crohn's Disease: An Underused Therapy

**DOI:** 10.1155/2013/482108

**Published:** 2013-12-05

**Authors:** S. Kansal, J. Wagner, C. D. Kirkwood, A. G. Catto-Smith

**Affiliations:** ^1^Enteric Virus Group, Murdoch Children's Research Institute, 462 Burwood Road, Hawthorn, Melbourne, VIC 3122, Australia; ^2^Department of Gastroenterology & Clinical Nutrition, Royal Children's Hospital, 50 Flemington Road, Parkville, Melbourne, VIC 3052, Australia; ^3^Department of Paediatrics, University of Melbourne, Australia; ^4^Department of Microbiology, Latrobe University, Melbourne, VIC, Australia

## Abstract

This paper reviews the literature on the history, efficacy, and putative mechanism of action of enteral nutrition for inflammatory bowel disease in both paediatric and adult patients. It also analyses the reasoning behind the low popularity of exclusive enteral nutrition in clinical practice despite the benefits and safety profile.

## 1. Introduction

Inflammatory bowel disease (IBD) comprising Crohn's Disease (CD) and ulcerative colitis (UC) represents a major health care problem. It affects over 60,000 Australians, with a direct healthcare cost exceeding AUD$2.7 billion per year [1]. The Australian Crohn's and Colitis Association predicts that by 2020 the number of people affected with CD and UC will increase by 20–25%. IBD is more prevalent in westernised regions including Europe, Australia, and North America although a rising trend has been noticed in Asia, Africa, and South America [2].

IBD causes chronic, destructive inflammation of the gastrointestinal tract [3, 4]. It is a lifelong condition, characterised by frequent disease relapses and remissions. Although genetics, the environment, and microbiota all play a part, the exact cause of CD or UC remains unknown and cures are unavailable [4] Therapy is designed to induce prolonged remission, which can often be achieved by a combination of corticosteroids and immunosuppressants. Corticosteroids constitute a first line of treatment to manage the acute presentation and relapses [5]. Various immunomodulators including Azathioprine, Methotrexate, and thioguanine analogues are frequently used for maintenance therapy [5]. More recently biologic agents such as infliximab are being used in patients with both steroid refractory and dependant luminal disease and fistulising and extraintestinal CD [6]. Infliximab is an antitumour necrosis factor alpha (TNF*α*) chimeric antibody and acts by inhibiting the action of TNF*α* [6, 7].

Unfortunately most of these treatment options, though effective, come at a significant cost to the patient in terms of adverse effects. Corticosteroids are limited in their use by risk of infection, osteoporosis, hypertension, growth retardation, poor mucosal healing, and early relapses on cessation of therapy [8]. This is especially problematic in paediatric patients who may experience significant growth retardation and osteoporosis with steroid therapy [9–12]. Prolonged immune suppression with immunomodulators is also concerning owing to the risk of opportunistic infections besides problems with hematologic disorders [13]. Biologic agents are limited by loss of efficacy over time due to the development of antibodies, as well as a risk of local reactions, anaphylaxis, and vasculitis [6]. Moreover the reported risk of lymphomas especially in young adult males on concomitant Azathioprine and infliximab, albeit low, further limits their use [14, 15].

Exclusive enteral nutrition (EEN) is a nutritional therapy used for the treatment of Crohn's Disease [16]. In general terms, it is used for induction of remission and is achieved by a period of 6–8 weeks of exclusive liquid feeding with either elemental or polymeric formulae. The patients are not allowed to have any other dietary items except plain water and some beverages.

EEN offers little risk but appears to be relatively underused compared to other modalities, except in paediatric practice. The purpose of our review was to examine the history of its introduction to current practice, relative efficacy, and possible mechanisms of action. In order to carry out this review, we conducted a PubMed search using key words “enteral nutrition” and “inflammatory bowel disease” which revealed 732 publications. Of these 100 were found to be relevant with 50 providing useful information which were used in this report.

## 2. History of EEN 

Nutritional therapy for CD has been employed ever since the condition was first described in 1932 [17]. The earliest reports of the use of nutritional therapy indicate that it was used primarily as a means to improve nutrition in debilitated patients unfit for surgical management. This nutritional therapy was in the form of a high protein, high carbohydrate, low residue diet with additional iron and supplements for specific nutritional deficiencies [18–20]. Specific beneficial outcomes from nutritional therapy were viewed as being due to improvement in nutritional status and the possibility that nutritional therapy might have a direct therapeutic value was not considered. Corticosteroids became pivotal to pharmacological management with a high protein, high carbohydrate diet used as an adjunct to provide gut rest and improve the nutritional status [20, 21].

The possible efficacy of a nutritional-based therapy for direct treatment of CD was first reported by surgeons in the 1970s when Votik et al. [22] treated 13 patients with elemental formula 17 times over a period of 22 days. All but one of the patients tolerated the formula and demonstrated not only weight gain but there appeared to be an improvement in inflammatory indices.

Logan et al. [23] subsequently found evidence of diminished gut lymphocyte and protein loss when enteral feeds were used in patients with extensive small bowel disease. A subsequent controlled trial by O'Morain [24] suggested clinically equivalent or even superior efficacy of elemental feeds over steroids for adults with relapses of CD. A subsequent meta-analysis of a mixture of 8 trials involving 413 patients published up to 1993 suggested that enteral feeds were significantly inferior on an intention to treat basis compared to corticosteroids in producing a clinical remission (pooled odds ratio 0.35; 95% confidence intervals, 0.23–0.53) [25] Clinical response to enteral feeds ranged from 53 to 80% by 3–6 weeks. A large part of this difference related to feed intolerance, with tolerance improving to 87–100% in those studies where feeds were administered by nasogastric tube. However, even allowing for this the clinical response to corticosteroids was consistently better in all studies than enteral feeds. Additional potential benefits not included in this meta-analysis included effect on general nutritional status, mucosal healing, and bone health [26].

Further studies compared enteral feeds with each other or other modalities. A randomized trial involving 36 mostly adults compared total parenteral nutrition with an elemental diet, in the absence of additional pharmacologic therapy [27]. Both had similar efficacy which was measured as a fall in the Crohn's disease activity index (CDAI) to less than 150. However, the elemental diet was found to be cheaper, simpler, and safer. In the same publication, Jones et al. also reported their clinical experience with 77 patients from 16 to 65 yrs of age who tried to maintain remission with a personal exclusion diet. These patients excluded specific dietary elements based on symptoms and used elemental diet as a supplement. 26 patients from this group remained in remission for 2 yrs and 18 for at least 3 yrs. Three patients reported that their erythema nodosum could be controlled with diet, while two had a similar experience with their Crohn's colitis.

Navarro et al. [28] studied the efficacy of continuous exclusive enteral nutrition on 17 paediatric patients. These patients were administered a mixed formulation comprising peptides, mono-and oligosaccharides, and medium chain triglycerides through a nasogastric tube infused by a peristaltic pump. After 2 weeks they were commenced on a commercial formula Pregestimil (Mead Johnson). After 4 months of exclusive nutrition, small meals were progressively introduced providing 50% of the caloric intake. Fibres were completely excluded. Exclusive constant rate elemental nutrition (CREN) was maintained effectively from 2 to 7 months and CREN supplemented by oral nutrition was continued for 12 to 22 months. They found that EEN was well tolerated, safe, and effective in inducing remission in active CD based on clinical (symptom disappearance, growth improvement; onset of puberty) and lab criteria (serum albumin, iron, and haemoglobin). They also observed that it was beneficial in reducing steroid dependence in one of the patients in the study group who had steroid dependence.

More recently, Rubio et al (2011) observed that fractionated oral feeds are as efficacious as continuous enteral feeds in their retrospective review of 106 paediatric CD patients [29]. They observed no significant difference in compliance rates in the two groups and also in PCDAI and other lab parameters.

Morin et al (1982) reported their clinical experience with EEN in the form of continuous elemental nutrition in four children with moderate to severe CD poorly controlled with steroids [30]. These children received continuous EEN with no other form of treatment for 6 weeks. All patients had a complete remission of symptoms, improved nutritional status, and significant height and weight gain [30].

Zoli et al. (1997) demonstrated that elemental diet was as efficacious as steroids in inducing remission in a randomized controlled trial in a cohort of adult patients with active CD [31]. They also hypothesised that it is probably more effective in enhancing the nutritional status of the patients through restoration of intestinal permeability [32].

The efficacy of EEN on steroid resistance and dependence was also evaluated in 18 adult CD patients by Quintrec et al. [32]. Clinical remission and steroid withdrawal was noted in eleven patients. The authors concluded that EEN though efficacious in steroid dependent and steroid resistant CD did not prevent disease relapse.

Despite positive reports of the benefits of EEN with elemental formulae, patient acceptance remained poor and clinicians were reluctant to use it as a first line treatment option, in part because of its poor palatability.

Studies were conducted to investigate the possible use of more palatable polymeric formulae to improve nutritional status in patients with CD. Cosnes et al. [33] evaluated polymeric against elemental formula. Forty five adult patients with CD were divided into 3 cohorts. One group received EEN in the form of an elemental formula; the second group exclusively received a polymeric formula, while the third group received the same polymeric formula in addition to oral feeds. Interestingly, the nutritional result was the same irrespective of whether elemental or polymeric feeds were used. This study did not compare the efficacy of polymeric and elemental formulae on CD activity.

In a double blind randomised control trial Royall et al. [34] compared the success inducing clinical remission of amino acid based formulae with peptide based formulae in adults over 21 days with continuous nasojejunal feeds. They randomised 40 patients with active CD into 2 groups: one received Vivonex-TEN (Nestle Nutrition), an elemental formula, while the second group received peptide based formula, Peptamen (Nestle Nutrition). The two formulae differed in their fat content: three percent in the amino based formula versus 33 percent in the peptide based formula. Patients received no other pharmacotherapeutic agents, although 17 continued to receive low dose prednisolone. Remission was defined as a fall in CDAI to less than 150. They found that the rate of clinical remission in the two groups did not differ significantly (84% in amino acid group and 75% in peptide group).

Rigaud et al. [35] also found that elemental and polymeric formulae achieved similar remission rates (67–73%) in patients with active CD unresponsive to steroids and/or complicated by malnutrition. They conducted a prospective, randomized control trial on 30 adult patients with active CD. The patients were randomised into two groups: one group received elemental enteral nutrition and the second group received polymeric enteral nutrition. Similar remission rates with significant improvement in nutritional status was noted in both the groups indicating that polymeric enteral feeds are as efficacious as elemental enteral feeds in inducing remission in active CD.

Ludvigsson et al. [36] conducted a multicentre randomised control trial to compare the relative efficacy of elemental to polymeric formulae in children. Thirty-three paediatric patients with active CD were randomised to receive either elemental formula (E028E; Nutricia) or polymeric formula (Nutrison Standard; Nutricia). Remission was defined as a fall in paediatric Crohn's disease index (PCDAI) to less than 10 or a 40% decrease in PCDAI. Remission rates in the two groups were not significantly different (69% versus 82% at 6 weeks). However the patients in the polymeric formula group were found to have a better weight gain as compared to the group on elemental formula.

## 3. Current Practice of EEN

There is a wide variability in the formulations and clinical practice used in EEN. A recent survey by Whitten et al. [37] demonstrated the existence of at least 23 different formulations for EEN. There are also differences in duration of EEN (4–6 weeks versus 6–8 weeks), type of EEN (elemental versus polymeric), and method of reintroduction of feeds.

Current practice to induce remission in paediatric CD often necessitates EEN as a polymeric or elemental formula over a period of 6–8 weeks either orally or by a nasogastric tube [38]. Most of these are polymeric formulae (90%) but some gastroenterology units prefer to use elemental or semielemental formulae although these were reported to be less palatable than the more popular polymeric formulae. Polymeric formulae are reported to cost less and taste better. The choice of formula is probably also dictated by clinician experience, funding, and local availability [38]. There are no controlled trials that have studied the appropriate length of treatment, but current practice appears to be based on most studies reporting the occurrence of clinical remission within this period [35, 36, 38]. Small amounts of water or beverages are allowed in this period. At the end of the 6–8 week period of EEN a low residue diet is slowly introduced. There is a paucity of data on the best method and foods while reintroducing feeds. Nearly 50% of the international gastroenterology units surveyed by Whitten et al. preferred to reintroduce feeds with low residue food [37]. Some centres prefer to introduce one simple meal every 3-4 days, while some others prefer slow introduction of individual foods [37]. The volume of the enteral formula is reduced proportionately to the oral intake. In our own centre (Royal Children's Hospital, Melbourne) patients are recommended to start with white bread, pasta, and rice and avoid wholemeal products.

A Cochrane meta-analysis on comparison of elemental formulae based on fat content (low fat <20 g/1000 kCal versus >20 g/1000 kCal) did not demonstrate a significant difference in efficacy of enteral nutrition; however a nonsignificant trend favouring very low fat and very low long triglyceride concentration was identified [39].

There is also variability in the approach to concurrent use of other drugs with EEN. Most units reported concurrent use of amino salicylic acid formulations, while some others reported the use of immunosuppressants or infliximab [40, 41].

## 4. EEN versus Corticosteroids

Corticosteroids are considered the first line treatment for active CD. They have been shown to induce remission in most patients when administered for a period of 4–6 weeks [42]. There have been several studies demonstrating equivalent efficacy of EEN in inducing remission in active CD.

Ruuska et al. [43] conducted a randomised control trial comparing the efficacy of whole protein based formula with corticosteroids in 19 children over a period of 11 weeks. The children treated with EEN had a longer relapse free duration and better nutritional status when compared to children in the corticosteroid group. This study also demonstrated the effectiveness of EEN in the treatment of children suffering from a relapse.

However, in contrast a German meta-analysis reported similar efficacy of EEN and corticosteroids in inducing remission. Schwab et al. [26] reviewed 38 publications which included 571 patients who received nutritional therapy. Remission rates with elemental diet were found to be similar to polymeric formula and also to corticosteroids. However, they also noted that nutritional therapy was associated with high cost and poor patient acceptance.

A recent study by Soo et al. (2013) [44] on paediatric patients with active CD demonstrated that EEN is not only as efficacious as corticosteroids in inducing remission but also improved the bone mineral density of the patients. They did a retrospective chart review of 105 children with active CD. Thirty-six had received EEN and 69 had received corticosteroids as first line treatment. They noted a similar remission and relapse rate in the two groups but better bone mineral density in EEN group.

The most recent Cochrane meta-analysis [39] comparing efficacy of steroids to EEN in inducing remission which included six trials (192 patients received EEN and 160 received steroids) yielded a pooled odds ratio of 0.33 (95% confidence interval 0.21%–0.53%) favouring corticosteroid therapy.

However, a recent working group established by the North American Society for Pediatric Gastroenterology, Hepatology, and Nutrition has reevaluated past meta-analyses of enteral feeds compared to corticosteroids and identified some problems with their interpretation [38]. The exclusion of several paediatric trials for methodological reasons may have unbalanced the findings. These omitted studies had all either favoured EEN over corticosteroids for clinical efficacy or found that they were similar.

A meta-analysis of paediatric trials by Heuschkel et al. (2000) found that corticosteroids and EEN are equally efficacious in inducing remission in paediatric patients with active CD. They included five paediatric studies comprising 147 children [45, 46]. Better compliance with elemental and semielemental formulae in children was reported when compared to adults who had up to 40% drop out rate with elemental and semielemental formulae due to intolerance.

Another meta-analysis by Dziechciarz et al. [47] found that the remission rate with EEN was similar to corticosteroids in paediatric patients with active CD when results from 4 RCTs comprising 144 patients were pooled.

## 5. EEN and Relapse

A number of studies have reported a 60–70% relapse rate in patients treated with EEN within the first 12 months of diagnosis [48, 49]. Gorard et al. [48] conducted a randomized trial on 42 patients with active CD and confirmed a similar rate of remission in the patients receiving corticosteroids and those receiving EEN. However, they noted a higher rate of relapse at the end of twelve months in the patients treated with EEN when compared to those treated with corticosteroids. Recently Grogan et al. [50] published their data on a double blind RCT trial of enteral nutrition with two years followup. They compared the efficacy of polymeric enteral formula to elemental enteral formula and followed up their patients for two years. Children with only large bowel disease were excluded from the study. The patients received EEN for six weeks followed by introduction of normal diet according to the institute protocol. They found no significant difference in remission rates in elemental and polymeric groups. However they found a 68% relapse rate amongst their patients in both groups. Interestingly most of the patients (82%) chose to have EEN as treatment of their first relapse.

However, some studies have shown that if enteral nutrition is continued as a supplement to normal diet after an initial period of EEN prolonged remission can be achieved.

Wilchanski et al. [51] conducted a retrospective study to determine whether supplementary enteral nutrition after an initial period of exclusive enteral nutrition might prolong remission. They divided 65 patients who had gone into remission with an initial course of EEN for 4–6 weeks into two groups: one comprising patients who had chosen to continue supplementary feeding at night in addition to normal diet during the day and the second group comprising those who had declined any kind of supplementary enteral nutrition. Higher relapse rates at 6 and 12 months after initial treatment were found in the control cohort when compared to patients who continued supplementary enteral nutrition.

Esaki et al. [52] also noted that remission can be prolonged by supplementary enteral nutrition. They conducted a retrospective, single centre study on an adult cohort of patients with CD. Patients who had gone into remission following total par enteral nutrition (TPN) were divided into two groups on the basis of calories received from enteral nutrition. Those who received more than 1200 kcal from enteral nutrition comprised the enteral nutrition group, while those who received less than 1200 kcal from enteral nutrition comprised the nonenteral nutrition group. They found a significantly higher rate of recurrence in the non-EN group as compared to the EN group. They also noted that patients with penetrating CD and previous history of surgery were at a significantly greater risk of recurrence in the EN group.

## 6. EEN and Disease Location

Conflicting data regarding efficacy of EEN in relation to disease location have been reported. Some studies have shown a poor response in patients with colonic CD, while others have found remission with EEN to be independent of disease phenotype. Afzal et al. (2005) enrolled 65 paediatric patients and divided them into three groups based on disease distribution [53]. The first group comprised patients who had only ileal or small bowel disease, the second group had ileo-colonic disease and the third group had only colonic disease. All patients received polymeric enteric formula for 8 weeks. Response was evaluated as a fall in PCDAI to less than 20. They noted a significant improvement in all the three groups following treatment; however the clinical remission rate was significantly less in the colonic group as compared to the ileal and ileocolonic groups.

Buchanan et al. [54] reported the outcome of 110 patients who had received a primary course of exclusive enteral nutrition with polymeric formula (Modulen IBD; Nestle) for 8 weeks. A number of their patients were on mesalamine based products but none of them were on azathioprine or methotrexate. Eighty percent of their patients achieved clinical remission irrespective of the disease location along with significant improvement in weight and BMI-*Z* scores. However they did report a significantly poorer response in patients with isolated terminal ileum involvement.

Wong et al. [55] reported the outcome of three adolescent patients with significant perianal disease who were managed with exclusive enteral nutrition for eight weeks and had significant improvement in symptoms, PCDAI, and mucosal healing. They were subsequently managed with a combination of enteral nutrition and Methotrexate for acute exacerbations and sulphasalazine as maintenance therapy with good results.

## 7. Mechanism of Action of EEN

There are various schools of thought on the mechanism of action of EEN. Research has illustrated that EEN could act through various mechanisms to promote healing including via direct effect on mucosa, reduction of proinflammatory cytokines, alteration of gut microflora, and improvement of nutrition. We look at the available evidence for all of these mechanisms.

## 8. Mucosal Healing

There is emerging evidence that mucosal healing in CD is associated with an improved long term outcome and reduced complications and can even help alter the natural course of the disease [56, 57]. In early days of its use, all the benefits were attributed to improvement in nutrition in a malnourished patient. However, it soon became obvious that EEN had a direct anti-inflammatory action as evidenced by decrease in inflammatory cytokines and mucosal healing even before the nutritional benefits became apparent [58]. There is reasonable evidence that use of EEN is more likely to be followed by mucosal healing in a patient, but the exact mechanism of action is still a matter of debate. Corticosteroids by comparison have a limited impact on mucosal healing. A prospective multicentre trial conducted by Modigliani et al. [59] found that only 29% of patients in clinical remission also achieved endoscopic remission at the end of seven week treatment of acute CD with 1 mg/kg/day prednisolone treatment.

Borrelli et al. [60] conducted an open label RCT comparing the effect of polymeric formula to corticosteroids on clinical variables and mucosal healing. No significant difference in the number of patients going into remission was found; however, a significantly higher number of patients achieved mucosal healing (74%) with the polymeric formulae as compared to corticosteroids (33%).

More recently, Grover et al. [61] conducted an open label prospective study to evaluate the effect of EEN on mucosal healing. They offered EEN to 26 newly diagnosed CD children and evaluated their BMI *Z* score, PCDAI, lab parameters of inflammation, and endoscopic assessment before and after six weeks of treatment. They found that 84% patients achieved clinical remission, 76% achieved biochemical remission, 58% had good early endoscopic remission, and 21% had complete transmural remission of ileal CD.

Fell et al. [62] [examined mucosal healing and cytokines in a prospective study of 29 paediatric patients who received EEN for 8 weeks. They found that 79% achieved complete macroscopic and histological mucosal healing in the colon and terminal ileum. Proinflammatory cytokines were downregulated with a fall in tumour necrosis factor-*α* (TNF-*α*). There was a decline in ileal and colonic interleukin-1*β* m RNA together with a fall in interferon *γ* mRNA in the ileum and a rise in transforming growth factor *β* 1 mRNA.

## 9. Reduction in Inflammatory Cytokines

TNF-*α* is implicated in increased intestinal permeability and diminished tight junction integrity in CD patients [63, 64]. Nahidi et al. [63] demonstrated complete inhibition of TNF-*α* with nutrition therapy and biologic agents but only partial inhibition with corticosteroids. They exposed Caco-2 monolayers to TNF-*α* in the presence of polymeric formula, hydrocortisone, and infliximab. TNF-*α* increased monolayer permeability and decreased tight junction integrity which was reversed by the polymeric formula and infliximab. However, hydrocortisone only partially reversed increased membrane permeability.

Polymeric enteric formulae have also been shown to downregulate various inflammatory cytokines including interleukin-1*β* interleukin-8, interleukin-*γ* besides reduction in serum C-reactive protein (CRP), erythrocyte sedimentation rate (ESR), and tumour necrosis factor-*α* (TNF-*α*) [64, 65].

De Jong et al. [65]demonstrated in an in vitro model that polymeric formula acted directly on immortalised colonic enterocytes and reduced IL-8 production in response to proinflammatory cytokines. They also demonstrated that freeze thawing or boiling did not destroy the anti-inflammatory activity of the polymeric formula.

## 10. Alteration of Gut Microbiota

Several studies have suggested that alteration of intestinal microbiota contributes to the development of IBD [66–68]. According to the “dysbiosis” hypothesis a breakdown between the balance of good bacteria and harmful bacteria significantly contributes to the development of IBD [69]. It has been suggested that EEN produces an anti-inflammatory action by modifying the gut microbiota. There is a paucity of data on this aspect; however a few small studies have demonstrated a significant modification in diversity of *Eubacteria*, *Bacteroides, Prevotella,* and *Clostridium coccoides* groups following EEN treatment. Leach et al. [70] investigated changes in Eubacteria, *Bacteroides*, *Clostridium coccoides*, *Clostridium leptum,* and Bifidobacterium during and after EEN in paediatric patients. A significantly greater change in bacterial composition was found in patients with CD following EEN when compared to controls. These changes were maintained for 4 months and were associated with decreased inflammation.

A study conducted by Tjellstrom et al. [71] evaluated the effect of EEN on microflora in active CD. Faecal samples were collected from eighteen children with active CD and analysed for short chain fatty acids as a marker of gut microflora function. The results were compared to those obtained from twelve healthy teenagers. They found that 79% of the children responded positively to EEN by showing decreased levels of proinflammatory acetic acid and increased levels of anti-inflammatory butyric acid similar to levels in healthy controls.

Variation in gut microbiota was also demonstrated by Lionetti et al. [72] in their study on enteral nutrition in paediatric patients. Nine children with active CD were managed with polymeric enteral nutrition and faecal samples were collected every 2-3 weeks. These were then analysed by temperature gradient gel electrophoresis (TGGE) for biodiversity in bacterial composition and compared to five healthy controls. They noted that the healthy children showed a host specific stable bacterial profile. However the CD patients were found to have their own specific bacterial profile at the start of the treatment which varied greatly between subjects and required time to achieve stability during exclusive and partial enteral nutrition in each subject. The authors hypothesised that this variation in gut microbiota by enteral nutrition could be attributed to the prebiotic and low residue properties of enteral nutrition formulae.

## 11. Improvement in Nutrition

Exclusive enteral nutrition has been shown to have significant nutritional benefits besides inducing mucosal healing and reducing inflammation. Although the conventional pharmacological combined therapies for IBD are quite efficacious and induce prolonged remission, their effect on growth is controversial. Glucocorticoids have been shown to adversely impact linear growth by suppression of osteoblastogenesis and inhibition of chondrocyte proliferation and chondrocyte synthesis. EEN exerts a beneficial effect on growth by reversing the growth hormone resistance state [73–76].

In a study conducted by Whitten et al. [77] EEN was found to not only improve inflammatory markers but also improve serum markers of bone turnover suggesting an improvement in bone health. Twenty-three newly diagnosed children with CD were enrolled and administered EEN for 8 weeks. Evaluation of changes in inflammatory markers and serum markers of bone turnover including C terminal telopeptides of Type I collagen (CTX) and bone specific alkaline phosphatase (BAP) was conducted. The patients were found to have a significant improvement in inflammatory markers, fall in CTX levels, and increase in BAP levels suggesting an improvement in bone health.

There is limited information on the effect of EEN on micronutrients. Zinc and selenium supplements have been recommended for patients on long term treatment with enteral nutrition as most formulae are deficient in them according to a Japanese study that evaluated serum selenium and zinc levels in 31 patients on enteral nutrition as maintenance therapy [78–81].

In another study Akobeng et al. [82] found an improvement in selenium levels in patients receiving 4 weeks enteral nutrition but associated with a significant depletion of vitamins C and E despite the formula having apparently adequate vitamin content.

These two studies were, however, significantly different in terms of patient profile and the stage of the disease at which the analyses were performed. It is difficult to draw any all-encompassing conclusions on the effect of enteral nutrition on serum micronutrients.

## 12. EEN Adults

There has been a large difference in the uptake of EEN between paediatric and adult CD practice [38]. There are arguments that EEN may be more effective in children with CD but also that it advantages growth and maturation, issues that are not indicated in adults. Nevertheless, there is good evidence that EEN is an effective therapeutic intervention in adult CD patients.

A few studies have shown similar remission rates as corticosteroids in newly diagnosed CD independent of the preexisting nutritional status of the patients. In a randomized controlled trial, Gonzalez-Huix et al. [49] reported similar efficacy and no increase in relapse rates in patients treated with EEN. They conducted a study on 32 patients with active CD randomized to receive either prednisolone or EEN. Patients in both the groups achieved remission and also maintained remission for similar length of time. EEN was administered nasogastrically in a continuous fashion and was also reported to be well tolerated.

Other studies in adult patients have been less supportive or exposed problems with adherence and tolerance to EEN. In a randomized control trial conducted by Gorard et al. [48], 22 patients with newly diagnosed CD received EEN and 20 patients received prednisolone. Patients with gastric surgery and contraindications to steroids or already on steroids due to any reason were excluded. Forty-one percent of the patients in EEN group could not tolerate EEN either orally or naso-gastrically. At the end of the study period both the groups had similar remission rates, but patients treated with EEN had a cumulative probability of relapse of 0.67, while those treated with steroids had a probability of 0.28.

EEN in the form of elemental formula is the primary therapy for active and quiescent CD followed in Japan in adult patients. Several studies from Japan have indicated the efficacy of enteral nutrition in the management of CD. These have dealt with various aspects of management of CD. Watanabe et al. [83] demonstrated the utility of enteral nutrition in the management of active CD. They divided their patients into two groups; one group was administered more than 900 kcal/day of elemental formula and the second group was administered less than 900 kcal/day of elemental formula. They found that the group receiving more than 900 kcal/day of formula showed a significant improvement in the cumulative nonhospitalisation rate.

Yamamoto et al. [84, 85] demonstrated the efficacy of enteral nutrition in suppressing postoperative recurrence of CD. They recruited 40 consecutive patients who underwent ileal resection for CD and divided them into two groups. One group received enteral nutrition through a nasogastric tube at night and low fat food in the day time, while patients in the second group received neither nutritional therapy nor were advised any food restriction. They were followed for a period of five years. The cumulative recurrence incidence rate requiring infliximab therapy was significantly lower in the enteral nutrition group.

## 13. Maintenance of Remission

Elemental nutrition is emerging as a useful option for maintenance therapy. Wilchanski et al. [51] had earlier demonstrated the possible utility of partial EN in the maintenance of remission. They retrospectively divided a cohort of 65 paediatric patients who had received EEN for active CD into two groups: one included those who had elected to stay on partial enteral nutrition and the other comprising those who refused the supplemental nutrition. They found that relapse rates at 6 months and 12 months were significantly higher in the control group as compared to the group that elected to continue the nutritional supplement.

More recent studies have shown that enteral nutrition administered alone as 50% caloric requirement or in combination with infliximab could be a suitable, less toxic, and more acceptable alternative to currently available therapies like Azathioprine, Methotrexate, and 6-thioguanine. For example, Takagi et al. (2206) conducted a randomized control trial to evaluate the usefulness of half enteral nutrition as maintenance therapy [86]. They randomized 51 patients with CD in remission after treatment of acute phase with steroids, EEN, infliximab, or surgical intervention into two groups. One group received 50% of their daily caloric requirement as elemental diet and the rest as normal diet. The second group did not receive any maintenance therapy. A significantly lower relapse rate was noted in the patient group receiving 50% elemental nutrition, prompting the drug and safety board to recommend the discontinuation of the trial. This study clearly demonstrated the effectiveness of even 50% enteral nutrition in preventing relapses, but we cannot draw any conclusions on the superiority of enteral nutrition over the currently used therapies as this was not evaluated in this study.

A Cochrane meta-analysis [87] on the role of enteral nutrition for maintenance of remission has concluded that enteral nutrition may be effective for maintenance of remission in CD either alone or in conjunction with conventional therapy.

The value of enteral nutrition in improving the durability of response to biological agents has also been examined. A prospective clinical trial comparing the efficacy of maintaining remission with infliximab alone versus enteral nutrition with infliximab did not find any additional benefit from supplementary enteral feeding. Yamamoto et al. [85] prospectively recruited 56 patients who had received clinical remission with infliximab into two groups for maintenance therapy. One group comprising 32 patients received enteral nutrition at night in addition to 5 mg/kg infliximab every 8 weeks. The other group received only 5 mg/kg infliximab every eight weeks. There were no significant differences in the maintenance of clinical remission between the two groups.

However, a subsequent retrospective multicentre study also conducted in Japan [88] appeared to favour the use of elemental nutrition with infliximab in patients for maintenance of remission after induction with infliximab. Maintenance of remission at one year was examined in adult patients from seven centres who had gone into remission following infliximab therapy and were on infliximab maintenance therapy. They found a significantly higher remission rate in the group receiving infliximab with enteral nutrition as compared to the group of patients that did not receive enteral nutrition.

There are no paediatric studies on this aspect of enteral nutrition. Further studies are required to clarify the role of enteral nutrition in conjunction with other agents.

## 14. Poor Acceptability of EEN

Despite robust evidence of the utility of EEN in induction of remission in CD, it continues to be overlooked as a treatment option. There is a wide variability in its use worldwide. While 62% of European gastroenterologists use EEN as first line treatment for the management of active CD in paediatric CD only 4% of North American gastroenterologists use it [89]. The reasons for such wide variation are multiple and often varied. A very common perception amongst people who do not use it regularly is poor acceptability amongst their patients. It is believed that the monotony of the same formula for 4–6 weeks combined with restriction of daily meals may lead to poor compliance and hence compromise clinical results. However, most studies in both children and adults have reported good patient compliance.

There was an issue of palatability with elemental feeds but that seems to have been improved upon by the use of polymeric formula with no compromise in clinical outcome. Moreover various flavouring agents are allowed to make the formula more palatable.

Use of EEN is also related to the personal experience and training of clinicians involved in patient care. Clinicians who have been trained in a setting where EEN is used routinely are more likely to use it in their own practice as compared to those who have not.

Another reason for poor acceptability of enteral nutrition is the lack of a uniform protocol describing its use. There is still a paucity of data on the optimal duration of treatment, with variations in duration, 4 weeks or 6 weeks, foods to be allowed during therapy: only water or water, clear fluids, and beverages; method of reintroduction of food: low fat or low fibre or normal family meals; use in maintenance of remission: 50% supplement at night or none at all and also use in combination with other agents. As further research sheds more light on these aspects it is likely that the acceptability will also improve.

## 15. Conclusion

Enteral nutrition has come a long way since it was initially trialled in the 1970s. Multiple studies in multiple forms have reinforced the fact that given exclusively, polymeric enteral nutrition is an effective and safe option to induce remission in acute CD patients, especially children. A full understanding of the mechanism of action remains at best sketchy. There are direct anti-inflammatory effects on the gut epithelium, favourably altering the balance of pro- and anti-inflammatory cytokines and probably modifying the gut microbiota. EEN has also been found to be effective in postoperative setting. There is some debate regarding the efficacy of enteral nutrition in relation to disease location but at the least disease location may not be as critical as was once thought. A recent area of interest is the role of partial enteral nutrition in maintaining remission, for which there is some supportive evidence. Further research will no doubt further clarify additional roles and functions for enteral nutrition, but it is now indisputable that enteral nutrition can be safely used to induce clinical remission with an efficacy approaching that of steroids minus the adverse effects. The biggest single barrier to the success of enteral feeds lies in their poor palatability.

## Abbreviations

CD: Crohn's disease
CDAI: Crohn's disease activity index
EEN: Exclusive enteral nutrition
IBD: Inflammatory bowel disease
PCDAI: Paediatric Crohn's disease activity index
RCT: Randomized control trial
TNF-*α*: Tumor necrosis factor-*α*
UC: Ulcerative colitis.
